# Data set for describing the elaboration of a compatible Gateway-based co-expression vector set and supporting its validation

**DOI:** 10.1016/j.dib.2016.11.013

**Published:** 2016-11-10

**Authors:** Loubna Salim, Claire Feger, Didier Busso

**Affiliations:** Service de Biologie Moléculaire, Institut de Génétique et de Biologie Moléculaire et Cellulaire, CNRS/Inserm/Université de Strasbourg, 1 rue Laurent Fries, BP10142, 67404 Illkirch cedex, France

**Keywords:** Co-expression, Expression screening, Gateway, Protein complex, Recombinational cloning, Versatility

## Abstract

This article contains Supplementary Data including methods and figures that relate to the article entitled “Construction of a compatible Gateway-based co-expression vector set for expressing multiprotein complexes in *E. coli*” (L. Salim, C. Feger, D. Busso, 2016) [1] that describes the elaboration and the validation of a set of versatile compatible plasmids for co-expression studies in *Escherichia coli*.

Here, we describe experimental procedures for plasmid construction and recombinant protein expression. We give the list of the 33 (co)-expression plasmids encoding fluorescent protein and we show extensive experimental data obtained for all combinations tested for validating our vector set.

**Specifications Table**

**Table:** 

Subject area	Biology
More specific subject area	DNA recombinant techniques and nucleic acids
Type of data	Tables, microscopy images, figure
How data was acquired	MacroFluo macroscope and Mithras multimode microplate reader (Berthold technologies, Bad Wildbad, Germany)
Data format	Analyzed
Experimental factors	For imaging with the MacroFluo, transformed cells were replicated onto a nitrocellulose filter and protein expression was induced by IPTG. Cells were directly imaged on the filter with a light intensity of 50%, an exposure time of 0.5 s and an optical focus of 0.5x. For expressed protein quantification, individual liquid culture were normalized to an OD600nm = 1 and protein were quantified on the Mithras reader.
Experimental features	Transformed *E. coli* cells were imaged with the MacroFluo and the fluorescent protein expression level quantified on the Mithras multimode microplate reader
Data source location	N/A
Data accessibility	Data are supplied with this article

**Value of the data**

• The compatible vector sets have been designed in order to express protein complexes containing up to six proteins, one of them could be 6x histidine tagged at its N-terminus.
• The vector sets have been designed for rapid cloning whatever the vector. To have the benefit of the vector sets, the genes of interest is first cloned by Gateway in a pDONOR vector. For single expression, the gene under control of T7 promoter 1 is rapidly cloned by Gateway. For co-expression, the gene under control of T7 promoter 2 is cloned by restriction-ligation in one or several plasmids linearized by *Nde*I+*Bgl*II and the gene under control of T7 promoter 1 is then cloned by Gateway. For a rapid cloning, restriction-ligation cloning of the gene under control of T7 promoter 2 can be coupled with the Gateway reaction to transfer the gene under control of T7 promoter 1 in a one-tube reaction.
• Fig. 1 provides a detailed map of the vector set with unique restriction sites for researchers to rapidly set-up a cloning experiment as described above and to have the required restriction sites for modifying the vector sets according to researcher’s needs.
• The way the vectors have been elaborated allows versatility and rapid cloning for testing numerous combinations of proteins to be expressed.
• The data provide a solid background for researchers to focus only on their experimental data without wondering about potential bias due to the vector(s).

## 1 Data

Here, we share methods and experimental data that relate to the article entitled "Construction of a compatible Gateway-based co-expression vector set for expressing multiprotein complexes in E. coli" by L. Salim, C. Feger and D. Busso [1]. We describe the cloning procedure not only for elaborating each one of the vector but to clone gene target as well. We give a detailed snapshot of the cloning area and describe the restriction site(s) to be used to modify the set. We share the protocols used to quantify protein expression level by imaging and by fluorescent measurement. Finally, we detailed experimental data (image and quantification) for all combinations tested for the 33 (co)-expression plasmids constructed.

## 2 Experimental design, materials and methods

### 2.1 Materials

The sequences of oligonucleotides (Sigma Aldrich - St Louis, MO) were summarized in Table 1. All enzymes used were from New England Biolabs (Beverly, MA). The pETDuet-1, pACYCDuet-1 and pCDFDuet-1 plasmids were from Merck-Novagen, EMD Millipore (Darmstadt, Germany). The IPTG and antibiotics were from Sigma-Aldrich.

Overhang sequences required for ligation with Duet plasmids digested by *Nco*I and *Hind*III (M1H and M10 primers) were bolded. Both sites were restored. Overhang sequences required for ligation with Duet plasmids digested with *Nde*I and *Avr*II (M2 primers) were bolded. The *Avr*II site was not restored.

### 2.2 Vector design

**Fig. 1:**
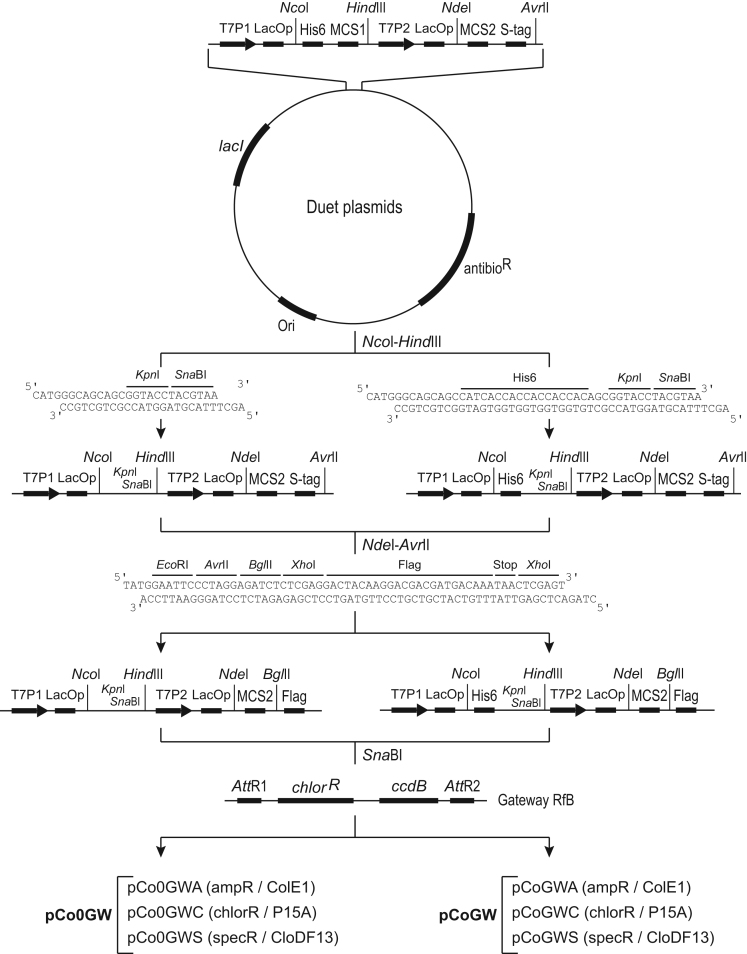
Design of pCoGW and pCo0GW vector sets. Briefly, the three selected pDuet plasmids were digested by *Nco*I+*Hind*III and primer hybrids were ligated. The resulting plasmids were digested by *Nde*I+*Avr*II and an additional primer hybrid was ligated. Finally, the six generated plasmids were linearized by *Sna*BI and the RfA Gateway cassette was ligated generating the pCoGW and pCo0GW vector sets. antibioR: gene conferring resistance to antibiotic (ampR: ampicillin, chlorR: chloramphenicol, specR: spectinomycin); His6: 6x histidine tag; LacOp: Lac operator; MCS: multiple cloning site; Ori: replication origin; T7P: T7 promoter.

The pETDuet-1, pACYCDuet-1 and pCDFDuet-1 plasmids were digested by *Nco*I+*Hind*III following manufacturer׳s recommendations. Digested plasmids were treated with shrimp alkaline phosphatase (5 U/µg of DNA) in digestion buffer for 15 min at 37 °C. The rSAP was inactivated by heat treatment at 65 °C for 15 min. One hundred picomoles of primers (see Table 1) were mixed by pair (M1_F with M1_R and M10_F with M10_R) and phosphorylated with T4 polynucleotide kinase (10 U) for 1 h at 37 °C in 1x PNK buffer. The T4 polynucleotide kinase was inactivated for 20 min at 90 °C. The annealing between complementary sequences of primer pairs occurred during cooling down to room temperature for about 30 min. Both primer hybrids (2 pmol) were ligated with digested plasmids (150 ng) with T4 DNA ligase (200 U) for 3 h at room temperature. Ligation mixes (10 µl) transformed 50 µl of home-made DH5α-T1R chemical competent cells [2] and positive clones were selected on appropriate selective agar plate. The inserted hybrid of primers brings an *Acc*65I/*Kpn*I site that can be used for further N-terminal tag addition and a *Sna*BI site for Gateway cassette insertion. After validation by DNA sequencing, the resulting plasmids were digested by *Nde*I+*Avr*II and a hybrid of phosphorylated primers (M2_F with M2_R) was ligated as described above. This second hybrid introduces a few restriction sites (*Nde*I, *Eco*RI, *Avr*II, *Bgl*II) as well as the sequence encoding a C-ter Flag tag. This sequence is flanked by an *Xho*I restriction site allowing replacement of the Flag tag by other tag encoding sequence if suited. Finally, the six resulting plasmids were digested by *Sna*BI in order to ligate the Gateway conversion cassette (RfA - Invitrogen, Carlsbad, CA) at the MCS1 following manufacturer׳s instructions. The resulting pCoGW and pCo0GW vector sets (Fig. 1) were sequence verified.

### 2.3 Cloning of fluorescent protein encoding genes

Gene encoding GFPuv (from pcDNA-DEST53 plasmid – Invitrogen), DsRed (from pDsRed2-N1 plasmid – Clontech, Mountain View, CA) and ECFP (from pECFP-C1 plasmid – Clontech), were amplified by specific primers. Forward primer contained the following sequence: four guanines (G) followed by the 25 nucleotide *att*B1 sequence (ACAAGTTTGTACAAAAAAGCAGGCT), two extra nucleotides to maintain the reading frame, sequence encoding TEV protease recognition site, the *Nde*I restriction site (CATATG), and at least an 18 to 25 nucleotides gene-specific sequence with a *Tm*=60 °C. Reverse primer contained the following sequence: four guanines (G) followed by the 25 nucleotide *att*B2 sequence (ACCACTTTGTACAAGAAAGCTGGGT), one extra nucleotide to maintain the reading frame once the LR recombination reaction occurred (important when the C-terminal Flag tag was suited), the *Bam*HI restriction site (GGATCC) and a Stop codon (when the C-terminal Flag tag was unwanted), and at least an 18 to 25 nucleotides gene-specific sequence with a *Tm*=60 °C.

The BP reaction was performed for 16 h at room temperature in a 5-µL reaction containing 1 µL of BP Clonase (Invitrogen) in 1x buffer plus 100 ng of gentamicin resistant pDONR207 plasmid and 30 fmol of PCR product purified on NucleoFast PCR plate (Macherey Nagel, Düren, Germany). After a 10-min incubation at 37 °C in the presence of 1.3 µg of proteinase K, 50 µL of DH5α-T1R chemically competent cells were transformed with the reaction and cells were plated on LB+gentamycin plates. Positive Entry clones were sequence validated and used to transfer the gene of interest either under control of T7 promoter 1 by Gateway cloning during the LR reaction or under control of T7 promoter 2 by restriction-ligation.

The LR reaction was performed for 16 h at room temperature in a final volume of 5 µL containing 1 µL of LR Clonase (Invitrogen) in 1x buffer with 100 ng of Entry clone plus 100 ng of pCoGW or pCoGW0 or pHGWA vector [3] as a control. After a 10-min incubation at 37 °C in the presence of 1.3 µg of proteinase K, 50 µL of DH5α-T1R chemically competent cells were transformed with the reaction and cells were plated on LB+antibiotic plates.

Restriction-ligation was performed by digesting 150 ng of pCoGW or pCo0GW plasmid by *Nde*I+*Bgl*II following manufacturer׳s recommendations. The digested plasmid was treated with shrimp alkaline phosphatase that was further inactivated as described in the previous section. The insert to be ligated was excised from the validated Entry clone by digestion with *Nde*I+*Bam*HI and a 1:5 M ratio of the insert was ligated in the digested plasmid as previously described.

All plasmids encoding fluorescent protein made for the study are listed in Table 2.

For the plasmids 1 to 21, the gene of interest under control of T7 promoter 1 was cloned in the appropriate plasmid by Gateway during the LR reaction. For the plasmids 22 to 33, the gene of interest under control of T7 promoter 2 was cloned by restriction-ligation and the gene under control of T7 promoter 1 by Gateway during the LR reaction.

N.A.: non-appropriate, T7.1: gene under control of T7 promoter 1, T7.2: gene under control of T7 promoter 2.

### 2.4 Cloning strategies

To have the possibility of screening for several protein partners for a given protein, the gene encoding the target protein could be inserted first under control of the T7 promoter 2 by restriction-ligation or by single-strand annealing using sequence and ligation independent cloning [4] to circumvent PCR product digestion. This initial cloning step should be performed in CcdB resistant strain such as DB3.1 or CcdB survival strain (Invitrogen) since the Gateway conversion cassette was still present. Thus, the resulting plasmid could be used for parallel LR reactions as described above to subclone easily the different protein encoding genes to be tested.

Furthermore, for a rapid cloning of two genes, the ligation/annealing of the gene under control of T7 promoter 2 might be performed first immediately followed, in the same tube, by the LR reaction to insert the gene under control of T7 promoter 1.

### 2.5 Protein expression and quantification

Expression vector(s) to be tested (50 ng) transformed 25 µl of BL21(DE3) chemically competent cells and transformation mix was split onto two selective agar plates. After an overnight culture, colonies obtained on one plate were replicated on a nitrocellulose filter and placed on a selective plate containing 1 mM IPTG for gene expression induction. The plate was incubated for an additional day at room temperature and imaged using the MacroFluo macroscope upon excitation using band-pass filters with the appropriate wavelength for the fluorescent protein expression level to be visualized (450–490 nm for GFP, 530–560 nm for DsRed and 416–436 nm for ECFP). All images were taken with a light intensity of 50%, an exposure time of 0.5 s and an optical focus of 0.5x.

In parallel, four individual colonies from the second plate were picked to inoculate individual starter culture (0.5 ml in 2xLB medium) supplemented with 2% glucose and the appropriate antibiotic(s) in 96-deep well culture plate for over-night at 37 °C. The day after, 50 µl of the starter culture were used to inoculate 2.5 ml of ZYM5052 auto-inducible medium [5] dispensed in a 24-deep well plate. Cultures were grown for 24 h at 25 °C under shaking at 350 RPM using a Microtron incubator-shaker (Infors, Basel, Switzerland). Upon cultivation, the biomass was determined by absorbance measurement at OD_600nm_ using the Mithras multimode microplate reader (Berthold technologies, Bad Wildbad, Germany) and protein expression was calculated by measuring emission fluorescence level (507 nm, 586 nm and 477 nm for GFP, DsRed and ECFP, respectively) upon excitation with the appropriate wavelength for GFP, DsRed and ECFP (385 nm, 556 nm and 434 nm, respectively). As negative control, liquid cultures were performed as previously described for strain transformed with the plasmid pHGWA-TtS1 encoding a non-fluorescent ribosomal protein. Values obtained with the negative control were 16312±341, 1357±23 and 12325±257 upon GFP, DsRed and ECFP excitation, respectively. The results displayed Table 3, Table 4 were normalized to an optical density of 1 and correspond to the mean±standard deviation for a triplicate experiment of 4 individual cultures per construct of the raw data minus blank.

Plates were imaged with the Macrofluo macroscope and protein expression level quantified with the Mithras as described in paragraph 1.5. Quantification results displayed correspond to the mean±standard deviation for a triplicate experiment of 4 individual cultures per construct of the raw data minus blank. The vector code is presented Table 2.

## Figures and Tables

**Table 1:** Oligonucleotides used for the construction of pCoGW and pCo0GW vector sets.

Name	Sequence (5′ to 3′)
M1H_F	**CATG**GGCAGCAGCCATCACCACCACCACCACAGCGGTACCTACGTAA
M1H_R	**AGCT**TTACGTAGGTACCGCTGTGGTGGTGGTGGTGATGGCTGCTGCC
M10_F	**CATG**GGCAGCAGCGGTACCTACGTAA
M10_R	**AGCT**TTACGTAGGTACCGCTGCTGCC
M2_F	**TA**TGGAATTCCCTAGGAGATCTCTCGAGGACTACAAGGACGACGATGACAAATAACTCGAGT
M2_R	**CTAG**ACTCGAGTTATTTGTCATCGTCGTCCTTGTAGTCCTCGAGAGATCTCCTAGGGAATTCCA

**Table 2:** List of constructed (co-)expression vectors.

#	Name	Code	T7.1	T7.2
1	pHGWA-GFP	pH-G	GFPuv	N.A.
2	pHGWA-DsRed	pH-R	DsRed	N.A.
3	pHGWA-ECFP	pH-C	ECFP	N.A.
4	pCoGWA-GFP	pCoA-G	GFPuv	None
5	pCoGWA-DsRed	pCoA-R	DsRed	None
6	pCoGWA-ECFP	pCoA-C	ECFP	None
7	pCoGWC-GFP	pCoC-G	GFPuv	None
8	pCoGWC-DsRed	pCoC-R	DsRed	None
9	pCoGWC-ECFP	pCoC-C	ECFP	None
10	pCoGWS-GFP	pCoS-G	GFPuv	None
11	pCoGWS-DsRed	pCoS-R	DsRed	None
12	pCoGWS-ECFP	pCoS-C	ECFP	None
13	pCo0GWA-GFP	pCo0A-G	GFPuv	None
14	pCo0GWA-DsRed	pCo0A-R	DsRed	None
15	pCo0GWA-ECFP	pCo0A-C	ECFP	None
16	pCo0GWC-GFP	pCo0C-G	GFPuv	None
17	pCo0GWC-DsRed	pCo0C-R	DsRed	None
18	pCo0GWC-ECFP	pCo0C-C	ECFP	None
19	pCo0GWS-GFP	pCo0S-G	GFPuv	None
20	pCo0GWS-DsRed	pCo0S-R	DsRed	None
21	pCo0GWS-ECFP	pCo0S-C	ECFP	None
22	pCoGWA-GFP-DsRed	pCoA-G+R	GFPuv	DsRed
23	pCoGWA-DsRed-GFP	pCoA-R+G	DsRed	GFPuv
24	pCoGWC-GFP-DsRed	pCoC-G+R	GFPuv	DsRed
25	pCoGWC-DsRed-GFP	pCoC-R+G	DsRed	GFPuv
26	pCoGWS-GFP-DsRed	pCoS-G+R	GFPuv	DsRed
27	pCoGWS-DsRed-GFP	pCoS-R+G	DsRed	GFPuv
28	pCo0GWA-GFP-DsRed	pCo0A-G+R	GFPuv	DsRed
29	pCo0GWA-DsRed-GFP	pCo0A-R+G	DsRed	GFPuv
30	pCo0GWC-GFP-DsRed	pCo0C-G+R	GFPuv	DsRed
31	pCo0GWC-DsRed-GFP	pCo0C-R+G	DsRed	GFPuv
32	pCo0GWS-GFP-DsRed	pCo0S-G+R	GFPuv	DsRed
33	pCo0GWS-DsRed-GFP	pCo0S-R+G	DsRed	GFPuv

**Table 3:** Imaging and quantification of single protein expression and co-expression from a unique plasmid.

**Table 4:** Imaging and quantification of (co)-expressed proteins from combination of plasmids.



